# Large vessel occlusion detection by non-contrast CT using artificial ıntelligence

**DOI:** 10.1007/s10072-024-07522-8

**Published:** 2024-04-15

**Authors:** Emrah Aytaç, Murat Gönen, Sinan Tatli, Ferhat Balgetir, Sengul Dogan, Turker Tuncer

**Affiliations:** 1 Department of Neurology, Faculty of Medicine, Fırat University, Elazig, Turkey; 2 Department of Digital Forensics Engineering, College of Technology, Fırat University, Elazig, Turkey

**Keywords:** Brain vessel occlusion, Computer vision, Machine learning

## Abstract

**Introduction:**

Computer vision models have been used to diagnose some disorders using computer tomography (CT) and magnetic resonance (MR) images. In this work, our objective is to detect large and small brain vessel occlusion using a deep feature engineering model in acute of ischemic stroke.

**Methods:**

We use our dataset. which contains 324 patient’s CT images with two classes; these classes are large and small brain vessel occlusion. We divided the collected image into horizontal and vertical patches. Then, pretrained AlexNet was utilized to extract deep features. Here, fc6 and fc7 (sixth and seventh fully connected layers) layers have been used to extract deep features from the created patches. The generated features from patches have been concatenated/merged to generate the final feature vector. In order to select the best combination from the generated final feature vector, an iterative selector (iterative neighborhood component analysis—INCA) has been used, and this selector has chosen 43 features. These 43 features have been used for classification. In the last phase, we used a kNN classifier with tenfold cross-validation.

**Results:**

By using 43 features and a kNN classifier, our AlexNet-based deep feature engineering model surprisingly attained 100% classification accuracy.

**Conclusion:**

The obtained perfect classification performance clearly demonstrated that our proposal could separate large and small brain vessel occlusion detection in non-contrast CT images. In this aspect, this model can assist neurology experts with the early recanalization chance.

## Introduction

Endovascular thrombectomy is the standard treatment developed in recent years for ischemic stroke patients with large vessel occlusion who experience symptoms within the first 6 h. [1]. The effectiveness of endovascular thrombectomy in acute ischemic stroke patients with large vessel occlusion is equal to time initially, and decreases with the narrowing of time according to the brain base [2]. Early treatment of patients with large vessel occlusion using endovascular thrombectomy reduces morbidity and mortality [3, 4]. Although endovascular thrombectomy is the primary treatment option for patients with large vessel occlusion, it is not available at every center. Therefore, the majority of these patients are initially evaluated at centers that do not perform thrombectomy. In this situation, difficulties arise in inter-hospital communication and referral to stroke centers for thrombectomy, in order to differentiate large vessel occlusion [5]. Even in experienced centers, it takes about 100 min to identify patients eligible for thrombectomy, which results in neuronal loss and an increase in morbidity [6]. In cases where reperfusion is achieved through endovascular thrombectomy, every 15-min delay results in a 5-point worsening of the NIHSS score [7]. Current guidelines recommend non-invasive imaging (such as computed tomography angiography/computed tomography perfusion/magnetic resonance angiography) to detect large vessel occlusion before angiography. They also state that any facility providing emergency stroke services should be capable of performing non-invasive vascular imaging [8]. However, due to resource constraints in stroke centers, multimodal CT imaging may not be sufficient [8, 9]. Acute access to MRI and CTP is not available in most centers in the USA or worldwide [10]. Less than one-third of small hospitals have 24/7 CT angiography available [11]. When vascular imaging is not feasible, NIHSS or the Rapid Arterial Occlusion Evaluation Scale have shown relatively high accuracy in detecting large vessel occlusion, but their level of adequacy is still not widely accepted [12]. According to recent clinical studies conducted in America and Europe, a combination of best medical treatment with endovascular thrombectomy has been reported to result in a cost savings of 33,190 pounds per patient compared to best medical treatment alone [13].

In this article, our primary goal is to propose a new automatic anterior system occlusion detection model based on computer vision to minimize human error. With the advancement of deep learning since the 2010s, computer vision models have been able to tackle various image classification problems with ease. Particularly, deep learning and patch-based models exhibit high accuracy rates and find application in many fields, such as biomedical image classification and object detection. To harness this capability of computer vision in urgent anterior system occlusion detection, we have suggested a patch-based transfer learning model. In our model, the image will be divided into 16 × 16 fixed-size patches. Features will be extracted from both the CT image and each fixed-size patch using a pretrained AlexNet. These extracted features will undergo iterative feature selection to identify the most meaningful attributes, and classifiers will be employed to obtain the results.

## Material and method

The study sample consisted of 324 patients hospitalized from the emergency department of Fırat University Hospital to the neurology clinic between January, 2019, and December, 2021, 159 of whom had large vessel occlusion and 165 of whom had small vessel occlusion confirmed by radiology report and clinical determination. Top of the ICA occlusion and MCA-M1 segment occlusion were included as large vessel occlusion. MCA-M3 and MCA-M4 distal branch occlusions were included as small vessel occlusions. Statistical analyses were performed using the SPSS package program version 25.0 (IBM, Armonk, NY). Descriptive data were reported as median (min–max). Chi-square test was used to compare categorical variables. Mann–Whitney U test was used to compare continuous variables between two independent groups. Statistical significance was accepted as *p* < 0.05 (Table 1). Sample size was calculated using G*Power software 3.1 with *α* = 0.05 and 1 − *β* = 0.95 [14]. When a study was not available, the minimum sample size was calculated as 314 by taking an effect size of 0.2.

**Table 1:** Baseline characteristic of the patients

	Large vessel occlusion (*n* = 159)	Small vessel occlusion (*n* = 165)	*p* value
Age	62 (37–79)	63 (39–78)	0.868
Gender (*n*,%)			
Female	74 (46.5)	81 (49.1)	0.646
Male	85 (53.5)	84 (50.9)	
Time from stroke onset, min [mean (min–max)]	194 (55–360)	209 (45–375)	0.555
NIHSS score [mean (min–max)]	16 (8–24)	15 (7–25)	**0.045**
ASPECT score [mean (min–max)]	8 (6–10)	8 (7–10)	0.134

*NIHSS*, National Institutes of Health Stroke Scale; *ASPEST*, The Alberta stroke programme early computed Tomography

Faul F, Erdfelder E, Buchner A, Lang AG. Statistical power analyses using G*Power 3.1: tests for correlation and regression analyses. Behav Res Methods. 2009 Nov;41(4):1149–60.

**Fig. 1:**
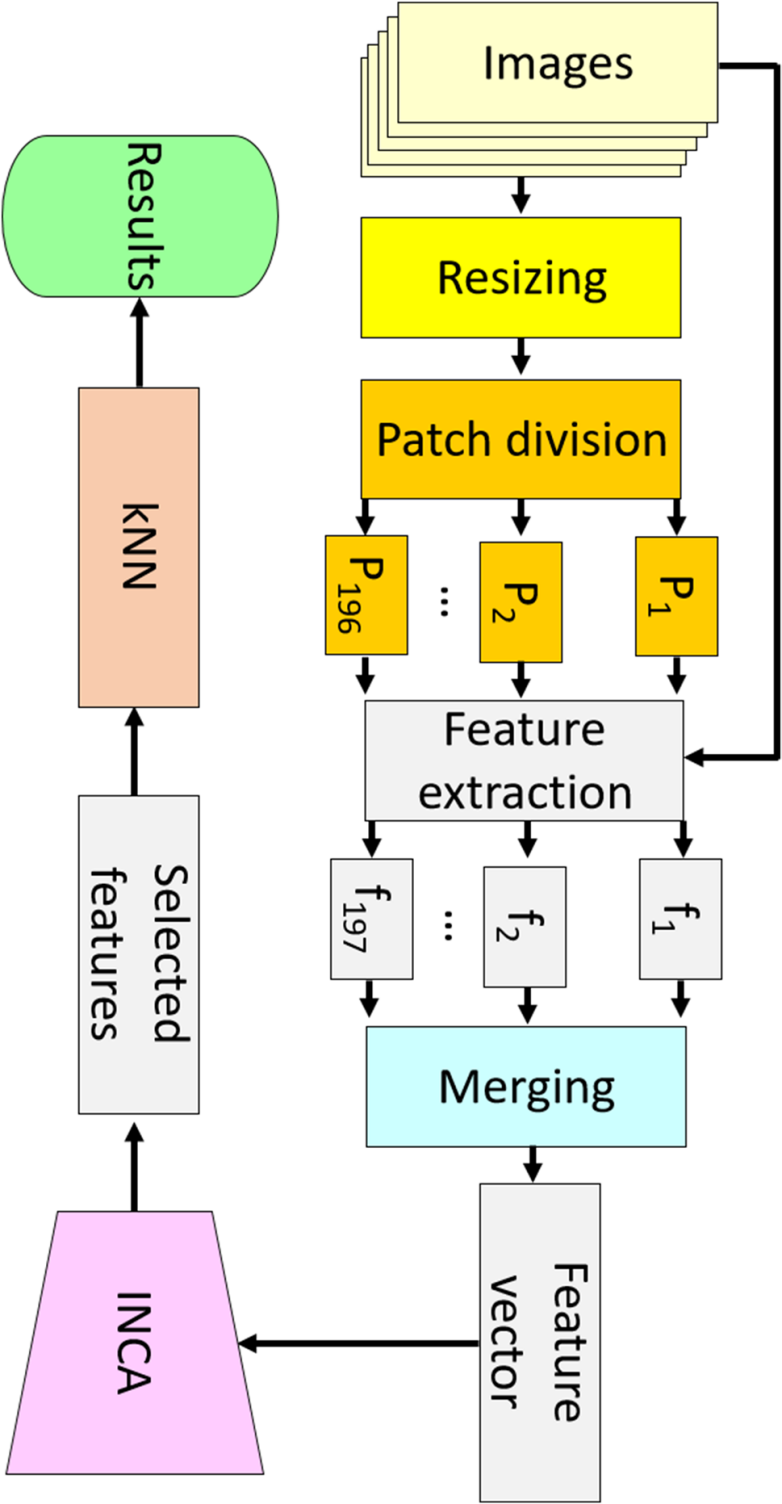
The block diagram of the proposed model

A new CT image classification model has been proposed in this work. We have presented a new patch-based (exemplar) deep feature engineering (PDFE) model. Our proposed PDFE model contains three essential phases, and they are (i) exemplar deep feature extraction, (ii) iterative feature selection, and (iii) classification with a kNN classifier. This model is a vision transformer (ViT) [15] like a model. In the ViT, features are extracted using a transformer from 16 × 16 sized patches. Here, we extracted features by deploying pretrained AlexNet [16]. We have used two fully connected layers of the AlexNet to get features named fc6 and fc7 (sixth and seventh fully connected layers). In this aspect, transfer learning has been used to get features from a patch of the image. By using this strategy, deep features have been extracted from each patch. In the feature concatenation phase, the generated features have been merged. To select the most informative/meaningful features, INCA [17] feature selector has been used. In the classification phase, the selected features have been fed to the classifier (kNN) [18]. The block diagram of the presented AlexNet-based PDFE is shown in Fig. 1.

The steps of the presented model have been listed below.

- Step 1: Read each image from the collected dataset.
- Step 2: Resize each image to 224 × 224-sized images.
- Step 3: Divide the resized image into 16 × 16-sized patches.
- Step 4: Generate a feature from the raw image by deploying the fc6 and fc7 layers of the pretrained AlexNet.
- Step 5: Extract deep features from each patch.

**Fig. 2:**
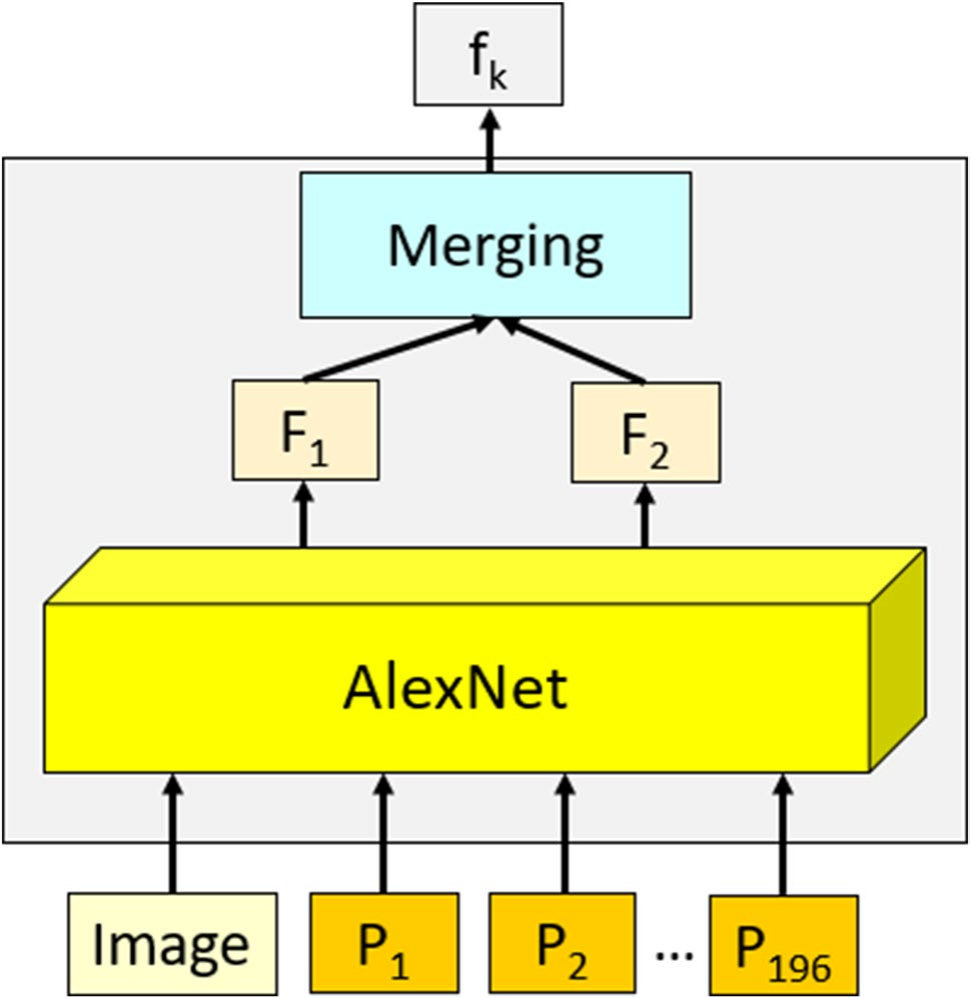
Feature extraction methodology of the proposed model. The raw image and the created patches (P) have been utilized as input of the pretrained AlexNet. The used AlexNet was trained on the ImageNet1K dataset. By deploying fc6 and fc7, two feature vectors, which are F1 and F2, are extracted, and the length of each feature vector is equal to 4096. These features have been merged, and 8192 (= 4096 + 4096) features (the created feature is demonstrated using f) are created from each input

The feature extraction phase (see step 4 and step 5) is demonstrated in Fig. 2.

- Step 6: Merge the generated features.
- Step 7: Apply INCA to the generated features and choose the most informative features.
- Step 8: Utilize the selected features (the selected 43 features) as input for the kNN classifier.

## Results

We proposed a new PDFE model using the pretrained AlexNet, INCA selector, and kNN classifier. To construct this model, we used MATLAB (2021a) programming environment. In the first phase, the pretrained AlexNet was imported to MATLAB by using the Add-Ons section. Our proposal was coded using functions. The coded functions are (i) main function, (ii) INCA, and (iii) kNN. By using the main function, AlexNet-based feature extraction was implemented. We have used the default settings of AlexNet. Furthermore, image resizing and patch division were coded in the main function. This function is also called INCA and kNN functions. In the meaningful feature selection phase, we have used INCA, and this feature selector is a parametric function. These parameters are given as follows. The loop range is from 1 to 500, and the loss function calculator is kNN. kNN has used both misclassification rate generators for feature selection and in the classification phase. Hyperparameters of this classifier are *k*, 1; distance, Euclidean; voting, none. Using the pretrained AlexNet, this model can be applied on a simple configured personal computer since there is no need to train AlexNet.

Classification accuracy, recall, precision, and F1-score have been used to evaluate the performance values, and the mathematical explanation of these parameters is given below [19, 20].

1 $$Accuracy=\frac{TP+TN}{TP+TN+FP+FN}$$

2 $$Recall=\frac{TP}{TP+FN}$$

3 $$Precision=\frac{TP}{TP+FP}$$

4 $$F1-score=\frac{2TP}{2TP+FN+FP}$$

The meanings of the used variables are $TP$, true positive; $TN$, true negative; $FP$, false positive; and $FN$, false negative.

Our proposal is applied to the collected dataset, and this dataset contains 324 CT images with two classes, and these classes are named (1) large occlusion and (2) small occlusion. There are 159 large occlusion CT images and 165 small occlusion CT images. Our model attained 100% classification performance, and the calculated performances are given in Table 2.

**Table 2:** Results (%) of the presented model

Evaluation metric	Result
Accuracy	100.0
Recall	100.0
Precision	100.0
F1-score	100

**Fig. 3:**
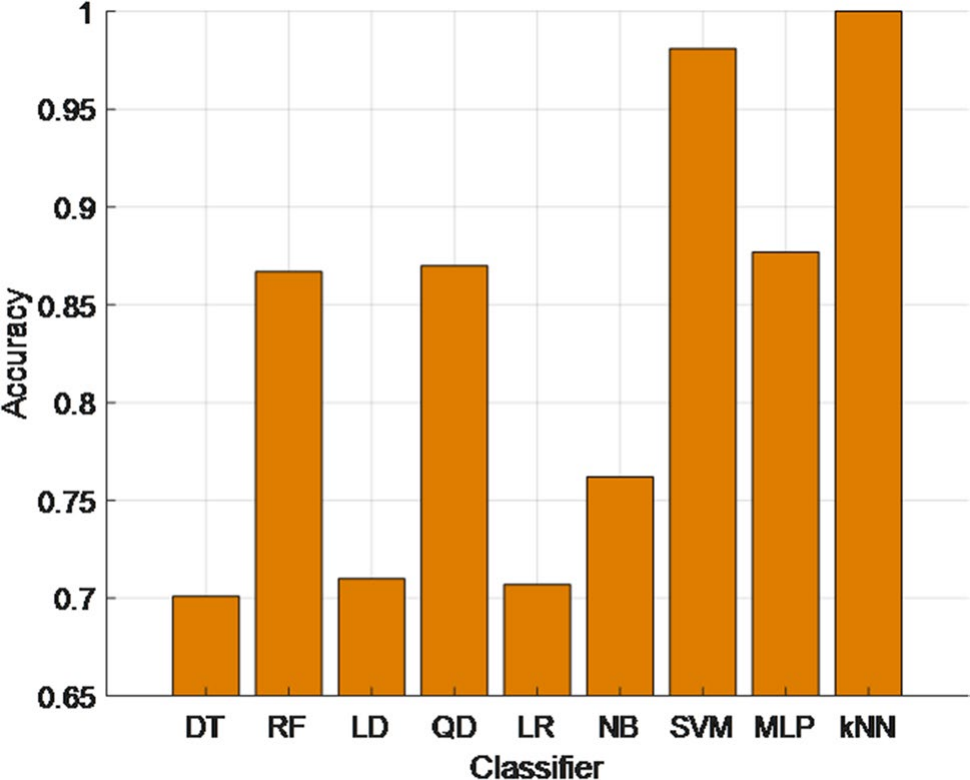
The classification performances of the used nine classifiers

As highlighted in Table 2, our model attained a wonderful classification performance by using kNN. In order to create a benchmark list, we have used decision tree (DT) [21], random forest (RF) [22], linear discriminant (LD) [23], quadratic discriminant (QD) [24], logistic regression (LR) [25], naïve Bayes (NB) [26], support vector machine (SVM) [27], and multi-layer perceptron (MLP) [28] for classification and the calculated classification performances are demonstrated in Fig. 3.

As can be seen from Fig. 3, the best classifier to solve this problem is kNN. SVM attained over 98.14% classification accuracy, and it is the second-best classifier for this model. The worst classifier is DT since it attained about 70% (DT reached 70.06% classification accuracy) classification accuracy.

## Discussions

Artificial intelligence (AI) is a general term that refers to the use of a computer to model intelligent behavior with minimal human intervention. Deep learning has begun to play a role in many aspects of medicine, from knowledge management to the control of health management systems, including electronic health records, and from doctors’ treatment decisions to active guidance [14–29]. Radiology emerged with the extraordinary discovery of X-rays, and since then, new imaging modalities (ultrasound, CT, MRI, PET, SPECT) have developed rapidly. Artificial intelligence is another such development that will potentially bring fundamental changes to the practice of radiology [15–30]. However, despite such rapid advances in radiology, it is not possible to distinguish between large and small vascular occlusions using brain CT images. We hypothesize that by utilizing artificial intelligence in brain CT imaging, we will be able to differentiate between large and small vascular occlusions in a fast, safe, side-effect-free and inexpensive manner.

For this purpose, we used various VIT models and evaluated the performance of each model in the classification of CT imaging. It has been observed that performing CT angiography to demonstrate the presence of large vessel occlusion can lead to contrast nephropathy, particularly more pronounced in individuals with diabetes mellitus. The additional cost incurred by performing CT angiography and the rapid loss of 1.9 million neurons, 14 billion synapses, and 12 km (7.5 miles) of myelinated fibers in the brain for every minute of large vessel occlusion have been determined [31, 32]. Therefore, achieving an accurate diagnosis promptly holds significant importance for the patient’s well-being. To handle this problem, machine learning is a well-known problem-solving methodology. Thus, we have presented a new transfer learning-based model.

In this study, we introduced a novel PDFE model utilizing the pretrained AlexNet architecture, the INCA feature selector, and the kNN classifier. Our approach involved three main coded functions: the main function, INCA, and kNN. The main function facilitated AlexNet-based feature extraction, incorporating default settings for AlexNet, image resizing, and patch division. This function was instrumental in integrating the INCA and kNN functions [17, 18]. We have applied the proposed PDFE to the collected CT image dataset for large- and small-occlusion detection. Our model attained 100% classification performance for solving this problem.

Based on these findings, using a CT image classification model as an alternative to CT angiography for detecting large vessel occlusion and considering the comprehensive evaluation of all structures, including deep brain structures, in this model, can yield positive results. Therefore, this assumption explains the outstanding classification performance of kNN models utilizing the CT image classification model.

We have discussed the most important points of this work and these are as follows:

- By proposing a transfer learning-based model, we have obtained a computationally cheap model. Therefore, we used a simple configured computer.
- We have used INCA to choose the most informative features automatically.
- Our model attained high classification performances using the shallow classifiers.
- Our model has a remarkable 100% accuracy, recall, precision, and F1-score, demonstrating exceptional capabilities in anterior system occlusion detection using kNN classifier.
- Our model has contributed to timely diagnosis by facilitating the swift identification of large vessel occlusion, thereby emphasizing its significance for patient well-being.

## Limitations and future works

We collected a CT image dataset from a single medical center, resulting in a relatively small dataset. However, we are actively working on obtaining a larger and more diverse dataset in the near future. Our current focus has been on the development of a CT image-based detection model. Additionally, we plan to incorporate CT angiography images and MRIs to expand the range of modalities. This will pave the way for a more comprehensive model in the near future.

Our primary objective in the near future is to validate our proposal in clinical settings. We intend to achieve this by assessing a wide array of patient populations and occlusion cases, ultimately confirming the practical utility and effectiveness of our machine learning-based model in real-world scenarios.

## Conclusions

In this study, we introduced a novel approach for detecting large vessel occlusion in ischemic cerebrovascular diseases using a PDFE model. By deploying the power of pretrained AlexNet, INCA feature selection, and the kNN classifier, we achieved outstanding results in identifying large vessel occlusion from brain CT images.

Our proposed PDFE model demonstrated exceptional classification performance, attaining perfect accuracy, recall, precision, and F1-score on a dataset containing two classes: large occlusion and small occlusion. Our proposal achieved 100% classification performances.

Our PDFE model demonstrates the significant strides that can be achieved by combining advanced machine learning techniques with medical imaging. The promising results obtained lay the foundation for future advancements and underscore the potential of technology-driven solutions in the realm of medical diagnosis and intervention.

## Data Availability

None.
